# Combinational administration of mesenchymal stem cell-derived exosomes and metformin reduces inflammatory responses in an in vitro model of insulin resistance in HepG2 cells

**DOI:** 10.1016/j.heliyon.2023.e15489

**Published:** 2023-04-14

**Authors:** Kosar Malekpour, Ali Hazrati, Sara Soudi, Leila Roshangar, Ali Akbar Pourfathollah, Majid Ahmadi

**Affiliations:** a Department of Immunology, Faculty of Medical Sciences, Tarbiat Modares University, Tehran, Iran; b Department of Immunology, School of Medicine, Tehran University of Medical Sciences, Tehran, Iran; c Stem Cell Research Center, Tabriz University of Medical Sciences, Tabriz, Iran

**Keywords:** Mesenchymal stem cell, Exosome, Diabetes, Insulin resistance, Inflammation, Immunomodulation

## Abstract

Diabetes is a highly common metabolic disorder in advanced societies. One of the causes of diabetes is insulin resistance, which is associated with a loss of sensitivity to insulin-sensitive cells. Insulin resistance develops in the body of a person prone to diabetes many years before diabetes development. Insulin resistance is associated with complications such as hyperglycemia, hyperlipidemia, and compensatory hyperinsulinemia and causes liver inflammation, which, if left untreated, can lead to cirrhosis, fibrosis, and even liver cancer. Metformin is the first line of treatment for patients with diabetes, which lowers blood sugar and increases insulin sensitivity by inhibiting gluconeogenesis in liver cells. The use of metformin has side effects, including a metallic taste in the mouth, vomiting, nausea, diarrhea, and upset stomach. For this reason, other treatments, along with metformin, are being developed. Considering the anti-inflammatory role of mesenchymal stem cells (MSCs) derived exosomes, their use seems to help improve liver tissue function and prevent damage caused by inflammation. This study investigated the anti-inflammatory effect of Wharton's jelly MSCs derived exosomes in combination with metformin in the HepG2 cells insulin resistance model induced by high glucose. This study showed that MSCs derived exosomes as an anti-inflammatory agent in combination with metformin could increase the therapeutic efficacy of metformin without needing to change metformin doses by decreasing inflammatory cytokines production, including IL-1, IL-6, and TNF-α and apoptosis in HepG2 cells.

## 1 Introduction

Type 2 diabetes (T2D) is a chronic inflammatory disease that results from increased blood glucose and lipid levels due to insufficient insulin production by β-pancreatic cells in response to different insulin concentrations [1]. Monocytes and macrophages residing in different tissues are the main sources of increased inflammatory mediators in T2D patients' serum [2]. However, non-immune cells in various tissues have been reported to produce different inflammatory cytokines locally, which helps accelerate tissue damage during T2D pathogenesis [3]. In this regard, it is believed that vascular endothelial cells, skeletal muscle cells, fatty cells, and liver cells (hepatocytes) release mediators by pro-inflammatory properties in response to various stimuli [4].

Insulin resistance occurs preliminary to T2D development and is associated with increased glucose production and fat accumulation in the liver [5]. Hepatocytes, by producing IL-1β, IL-6, and tumor necrosis factor (TNF-α) in response to stimulation by bacterial infection, lipopolysaccharides (LPS), hepatocyte growth factor (HGF), H2O2, and TNF-α are a source of inflammatory mediators in the liver [6]. It has also been shown that hyperglycemia as a consequence of insulin resistance is one of the factors influencing inflammation in different cell types [7]. Hyperglycemia exposure induces significant inflammatory responses in hepatocytes and adipocytes [8]. The combined action of mitochondrial oxidative stress, hyperinsulinemia, and hyperglycemia causes the formation of free radicals, which in turn induce inflammation and cell necrosis [9]. These extensive cell apoptosis in the liver can lead to an abnormal increase in liver enzymes, Nonalcoholic fatty liver (NAFLD), fibrosis, cirrhosis, and hepatocellular carcinoma (HCCs) [10]. As a consequence of the MAPKs and NF-κB signaling pathways activations, pro-inflammatory cytokines production, including TNF-α, IL6, and IL1-β, increases and leads to the recruitment of immune system cells (such as neutrophils and T lymphocytes) and triggers liver damage [11].

Metformin was derived in 1920 from Galega officinalis and has been used as a hypoglycemic drug since 1950. Metformin has a guanidine structure, positive charge, and hydrophilic properties and is the primary line of treatment for T2D [12]. The therapeutic function of metformin can be attributed to its interaction with several enzymes, including complex I mitochondrial electron transfer chain and AMP-activated protein kinase (AMPK) [12]. But due to the side effects of metformin, such as a metallic taste in the mouth, vomiting, nausea, diarrhea, and upset stomach. For this reason, other treatments along with metformin are being developed [13].

Studies show that mesenchymal stem cells (MSCs) can be used in cell therapy for liver disease [14]. MSCs can differentiate into hepatocyte-like cells, reduce liver inflammation, and prevent fibrosis by suppressing the function of hepatic satellite cells (HSCs) [15,16]. In addition, these cells can suppress the immune system responses and decrease hepatocytes apoptosis [17]. Nevertheless, iatrogenic tumor formation, cell rejection, and toxicity are risks in MSCs transplantations [18,19]. The advantages of MSCs-derived exosomes over MSCs application include lower toxicity, relative stability in the bloodstream, lower immune response, and ease of transfer to the target cell [18,20]. Also, because there is no use of living cells, there is no risk of developing tumors in their application [21]. The regenerative and immunomodulatory effects of MSC-derived exosomes in liver disease have been shown in recent studies [[22], [23], [24], [25]].

**Fig. 1:**
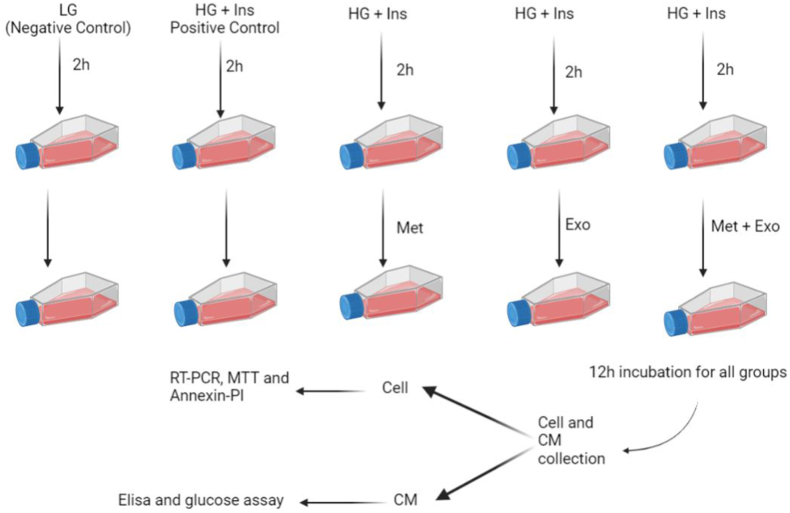
Study Design. This figure is created by Biorender.com.

In this study, to simulate the insulin resistance model in the liver of diabetic patients in vitro, the human hepatocellular carcinoma cell line (HepG2) was cultured in a high glucose-containing cell culture medium to induce inflammation. Metformin was used to increase insulin sensitivity as well as to reduce inflammation, and MSC-derived exosomes were used to enhance the anti-inflammatory effects of metformin (Fig. 1).

## 2 Material and methods

### 2.1 HepG2 cell culture

HepG2 cells were purchased from the Iranian biological resource center (IBRC, Tehran, Iran). They were maintained as an adherent cell line in DMEM (Dulbecco's Modified Eagle Medium) Low Glucose (Gibco, NY, USA) with 10% fetal bovine serum (FBS), 1 × nonessential amino acids, and 2 mmol/l l-glutamine at 37 °C in a 5% CO2:95% air-humidified incubator. When cells confluency rich 75–80%, they were passaged using 0.5% trypsin-EDTA (Gibco, NY, USA) for cells detaching from the bottom of the cell culture flask.

### 2.2 Determine the metformin dose and incubation period

To determine the metformin dose and incubation period for different treatment groups, the experimental groups were first divided into 12-h and 24-h groups. In each of the above groups, cells were cultured in a high glucose-containing DMEM medium with 30 mM glucose and 10^−7^ mM insulin. Then, after 2 h, different doses of metformin, including 0.1 mM, 0.5 mM, 1 mM, 2 mM, and 5 mM, were added to the culture medium. Then, the viability of treated cells was evaluated by MTT assay after 12 h of incubation. Cell viability was also assessed by Trypan Blue. The membrane of living cells does not allow the trypan blue dye to pass, so living cells are colorless and dead cells are blue under a light microscope.

### 2.3 Experimental groups

To evaluate the immunomodulatory and hypoglycemic effects of MSC-EXO, metformin, and their combinatory uses on HepG2 cells, these cells are cultured in 6 plate wells. Experimental groups were defined as (1) HepG2 cells + low glucose, (2) HepG2 + High glucose (HG) + Ins, (3) HepG2 + HG + Ins + Met, (4) HepG2 + HG + Ins + EXO, and (5) HepG2 + HG + Ins + EXO + Met. 24 h after cell passage and their transfer to plates, different treatments are applied to the cell culture medium. For this purpose, except for the control group containing low glucose (LG) medium (5 mM glucose), other groups were incubated for 2 h with (HG) medium (25 mM glucose) and insulin (10^−7^mM). Then various treatments were added to the experimental groups, including metformin (1 mM) and exosomes (100 μg/ml). Finally, after 12 h, the cells were used for MTT, Annexin-PI, and real-time PCR. In addition, the supernatant was collected 12 h after various treatments and used to assess cytokine levels (Elisa) and glucose concentrations.

### 2.4 Wj-MSCs isolation

The human umbilical cord tissue (hUC) was collected and then cut into 0.5–1 cm pieces. These pieces were washed with culture medium and PBS, and the three observed vessels, including two arteries and one vein, were removed carefully. Each piece was placed in 10 cm^2^ plates or 25 cm^2^ flasks. DMEM/F-12 (Gibco, Grand Island, NY), containing 15–20% FBS (Gibco, Grand Island, NY), was added to the plates and flasks. The cells' growth and expansion were monitored daily and passaged using 0.5% trypsin-EDTA when the confluence was about 80–85%.

### 2.5 MSCs characterization

The third passage MSCs isolated from WJ tissue surface marker expression were assessed by FACSCalibur flow cytometer (BD Biosciences, USA). Anti-human antibodies against CD73, CD34, CD31, and CD44 were used to evaluate the WJ-MSCs surface markers (all from eBioscience).

Considering that MSCs should have the ability to differentiate into other cells, also the WJ-MSCs were evaluated for their osteogenic and adipogenic differentiative ability. For this purpose, according to the previously performed protocol [25], the cells were cultured in the purchased differentiation culture mediums for three weeks, and their culture medium was regularly monitored. Then they were examined for osteogenic and adipogenic differentiation using Alizarin Red-S (ARS) and Oil Red-O (ORO) staining, respectively.

### 2.6 Exosome isolation

After culturing and expanding MSCs, the passage 2 or 3 supernatants were collected and exchanged with a lower FBS medium. The MSC culture medium is changed twice weekly with a lower FBS-containing culture medium. Using this steps, the MSCs were adapted to an FBS-free medium. Then MSCs cultured in an FBS-free medium for 72 h, and their supernatant was collected and were centrifuged. For removing cell debris, collected supernatants were filtered using 0.22 μm filters. Then, the pure exosomes were used for exosome characterization and exosome-treated groups.

Modified guidelines of the Exocib kit (Cib Biotech Co.) are used for exosome isolation from supernatants. After filtering supernatants, 15 ml were mixed with 3 ml of reagent A (viscose reagent) for exosome isolation. After incubation overnight at 4 °C, the tubes were centrifuged at 1000 g for 40 min. Finally, depending on the size of the pellet, 100–200 μl PBS was added to the pellet, and the mixture was resuspended. The concentration of the isolated exosomes was assessed using a Bicinchoninic Acid (BCA) kit (DNAbiotech Co.).

### 2.7 Exosome characterization

The isolated exosomes' (fixed with glutaraldehyde) shape and size were evaluated using scanning electron microscopy (SEM) (MIRA3 TESCAN). After washing and dehydrating with PBS and ethanol, the samples were analyzed by SEM. To measure the average size of exosomes, 10 μl of the different samples were diluted in PBS. After mixing samples, they were transferred into a cuvette and then analyzed by dynamic light scattering (DLS) (Malvern Instruments, UK). Transmission electron microscopy (TEM) (Zeiss-EM10C) was also used to characterize isolated exosomes shape. Exosomes were fixed in glutaraldehyde and paraformaldehyde to evaluate their shape by TEM.

### 2.8 Viability assessment in experimental groups

MTT test was used to evaluate the proliferation and survival of HepG2 cells in different treatment groups. For this purpose, the cells were cultured on plate 96 cells. Then they receive different treatments in triplicates. After 12 h of incubation, the supernatant was collected, and the cells were washed twice with PBS solution. Then 20 μl of MTT solution was added to each well, and the plate covered with aluminum foil was placed in a cell culture incubator for 4 h at 37 °C. At the end of the incubation time, the MTT solution was gently removed from the plate by an insulin syringe, and to each well, 100 μl DMSO was added. Finally, absorbance was read at 540 nm by spectrophotometer.

### 2.9 Glucose consumption assay

As described previously, the insulin resistance-induced HepG2 cells were treated with various metformin concentrations. The supernatant of each well was collected, and using a glucose assay kit (glucose oxidase method), glucose content was detected. The glucose consumption was calculated as follows: glucose consumption = 30 mmol/L - cell supernatant glucose content.

### 2.10 Real-time PCR gene expression quantification

HepG2 cells were exposed to the high glucose culture medium for 2 h. Then, treatments such as metformin alone, exosome alone, and the combination of Met and exosome are added to the HepG2 cells culture medium. Then the supernatant was collected, cells were washed two times with PBS, and using 0.5% trypsin-EDTA, cells were detached and collected. Total RNAs were isolated from experimental groups using the GeneAll RibospinTM total RNA purification kit (GeneAll Biotechnology, Seoul, South Korea). cDNA was synthesized by using 1 mg of total RNA and the RevertAid First Strand cDNA Synthesis Kit (Thermo Fisher, Northumberland, UK). Gene expression was measured by qRT-PCR using SYBR Green RealQ Plus 2 Master Mix Green (Ampliqon, Skovlunde, Denmark) on Corbett Rotor-Gene 6000 Light Cycler (Qiagen, Hilden, Germany). The primers are listed in Table 1. The obtained data were normalized by β-actin transcript level as a house kiping gene. The 2^–ΔΔCt^ method was used to calculate the relative gene expression. This study was performed independently 5 times on different groups of HepG2 cells in triplicates.

**Table 1:** The sequences of primers used in quantitative real-time polymerase chain reaction (qRT-PCR) assay.

	GEN	TM	Primer sequence
1	IL-1β	56	Forward: ACGATGCACCTGTACGATCA
			Reverse: TCTTTCAACACGCAGGACAG
2	TNF-α	60	Forward: GTCGTACAAACCACCAAGC
			Reverse: TGTGGGTGAGGAGCACATAG
3	IL-6	56	Forward: ACTCACCTCTTCAGAACGAATTG
			Reverse: CCATCTTTGGAAGGTTCAGGTTG
4	IL-10	54	Forward: CATCGATTTCTTCCCTGTGAA
			Reverse: TCTTGGAGCTTATTAAAGGCATTC

### 2.11 Measurement of cytokine concentration

HepG2 cells were exposed to the high glucose culture medium for 2 h. Then, treatments such as metformin alone, exosome alone, and the combination of metformin and exosome are added to the HepG2 cells culture medium. The supernatant of the different groups was collected and centrifuged for 5 min at 363 g to remove cell debris and detached cells and stored at −70 °C. Finally, the levels of IL-6, TNF-α, IL-1β, and IL-10 cytokines in the supernatant were measured by a commercial ELISA kit (R&D Systems, USA) according to the manufacturer's instructions. Each sample was dispensed in triplicate, and the optical density of each well was determined at 450 nm. This study was performed independently 5 times on different groups of HepG2 cells in triplicates.

### 2.12 Apoptosis assay

HepG2 cells were exposed to the high glucose culture medium for 2 h. Then, treatments such as metformin alone, exosome alone, and the combination of metformin and exosome are added to the HepG2 cells culture medium. BioLegend AnnexinV-PI kit was used to evaluate the percentage of apoptosis and necrosis in different treated groups (BioLegend, USA). At the end of the treatment and incubation period, the cells were removed from the bottom of the flask with 0.25% trypsin-EDTA (Sigma, USA). After neutralizing trypsin with 10% FBS containing DMEM medium, the cell suspension was centrifuged for 5 min at 1500 rpm. Then the supernatant was removed, and the cell pellet was dissolved in 500 μl binding buffer and centrifuged at 1500 rpm for 5 min. Then in the dark environment, 5 μl AnnexinV dye was added to each microtube and incubated for 20 min in the dark. Then, 5 μl of PI dye was added to the cell suspension and analyzed by FACSCanto II flow cytometry within 1 h. Flow cytometric data were also analyzed by Flowjo software. This study was performed independently 5 times on different groups of HepG2 cells in triplicates.

### 2.13 Statistical analysis

Graph Pad Prism 8.3.2 software (*t*-test and one-way ANOVA) is used for statistical analysis. The significance level was considered less than 0.05 (p < 0.05), and the data were expressed as Mean ± SD. Flow Jo software was also used to analyze flow cytometric data. In this study, P < 0.05 as *, P < 0.01 as **, P < 0.001 as *** and P < 0.0001 as ****.

## 3 Results

### 3.1 Metformin dose and incubation period determination

**Fig. 2:**
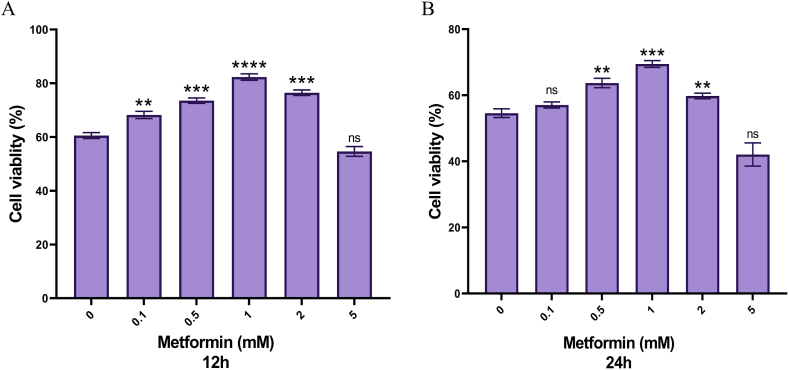
Evaluation of HepG2 cells viability in different doses of metformin in 12 and 24-h incubation.

As shown in the figure, the results of the MTT test showed that the 1 mM metformin doses and the12 h incubation period had better results and a higher survival rate than other doses and incubation times (Fig. 2A and B). As shown in Fig. 2, the cell viability in HepG2 cells witch treated with 5 mM of metformin was significantly reduced (P < 0.05).

### 3.2 WJ-MSC characterization

**Fig. 3:**
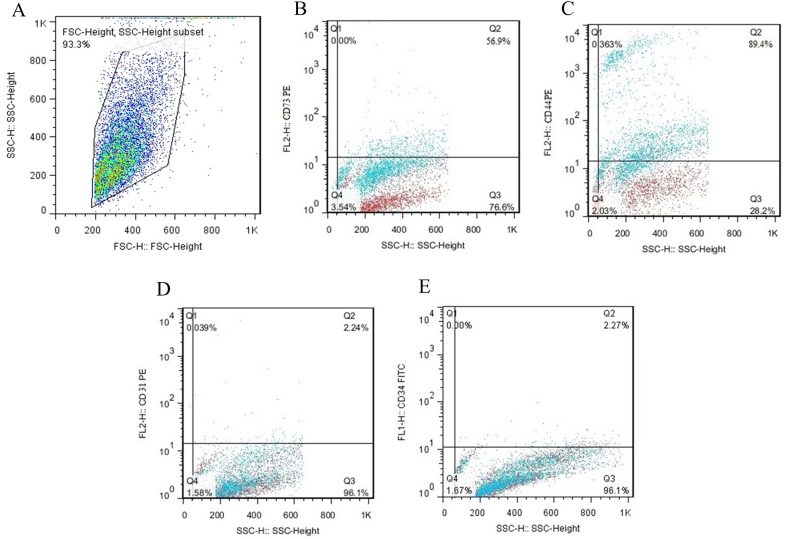
**Characterization of WJ-MSCs.** The expression of specific surface markers in WJ-MSCs includes CD73, CD31, CD34, and CD44 analysis by flow cytometry.

**Fig. 4:**
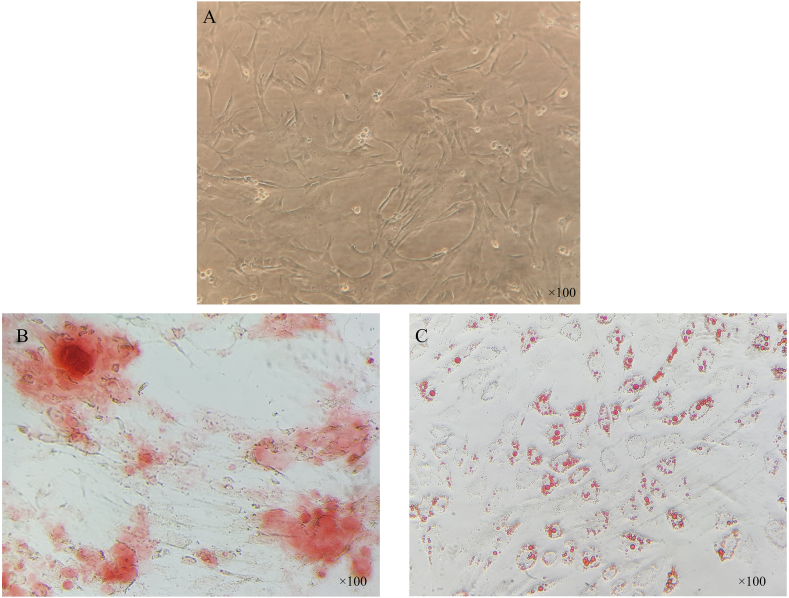
**WJ-MSCs differentiation potential**. (A) WJ-MSCs spindle fibroblast-like shape monitored by light microscopy. The differentiation potential of WJ-MSCs into (B) osteogenic and (C) adipogenic lineages was confirmed by Alizarin red S staining of calcium phosphate accumulation and Oil red O staining of lipid droplets, respectively.

Flow cytometry analysis showed that the majority of WJ-MSCs were positive for CD73 (56.9%) and CD44 (89.4%) markers (Fig. 3B&C) and for CD31 (2.27%) and CD34 (2.24%) markers were relatively negative (Figure (Fig. 3D&E). The spindle-like shape and proliferation of the cultured MSCs were monitored using an inverted microscope (Fig. 4A). In addition differentiation capacity of isolated MSCs, to the adipogenic and osteogenic lineages was evaluated. Calcium phosphate accumulation (osteogenic differentiation) was visualized after Alizarin Red-S and Oil Red-O staining, respectively (Fig. 4B and C).

### 3.3 WJ-MSC-EXO characterization

**Fig. 5:**
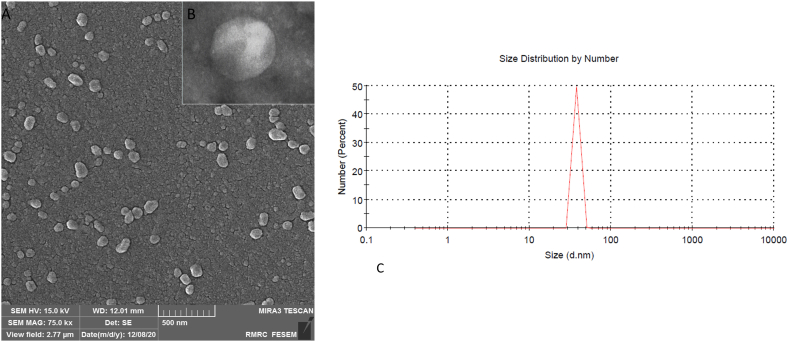
**Characterization of WJ-MSC-EXO.** (A) The shape of isolated exosomes was confirmed using scanning electron microscopy and (B) transient electron microscopy. (C) the average size of WJ-MSC-EXO was 44.21 nm as determined by dynamic light scattering (DLS).

SEM and TEM analysis visualized the isolated exosomes' shape and approximate size (Fig. 5A and B). DLS assay was performed to determine the precise size distribution of the exosomes. According to the DLS results, it was observed that the mean size of the isolated exosomes was 38.23 nm (Fig. 5C).

### 3.4 Evaluation of cytokines level in the supernatant

**Fig. 6:**
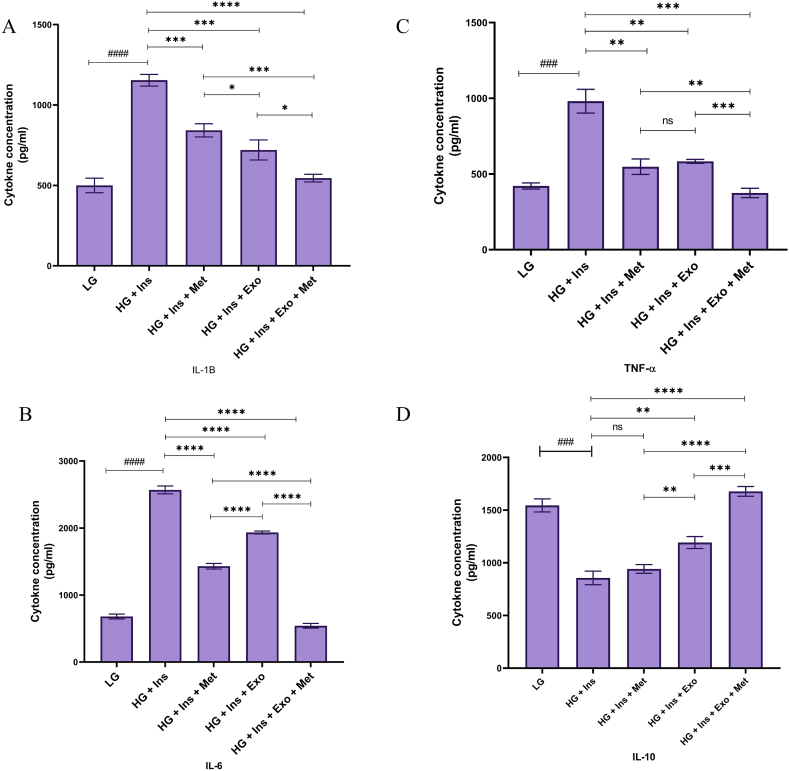
**The concentration of inflammatory cytokines (IL-6, IL-β, and TNF-α) and anti-inflammatory cytokine (IL-10).** The concentration of these cytokines in the supernatants of different experimental groups was measured using ELISA 12 h after treatment. Statistical analysis was performed by one-way ANOVA followed by Tukey's multiple comparison test. Results were presented as means ± standard deviation (SD). * indicates a P value of *<* 0.05, ** indicates a P value of *<* 0.01, *** indicates a P value of <0.001, and **** indicates a P value of <0.0001.

The concentration of IL-1β in the supernatant of LG cultured HepG2 cells was 502.32 ± 44.98 pg/ml, which increased to 1154.87 ± 36.32 pg/ml in the HG culture (P < 0.0001). The production of IL-1β in Met-treated HepG2 cells was 842.38 ± 41.020 pg/ml, which was significantly reduced compared to the group treated with HG DMEM (P = 0.0006). Also, the production of this cytokine in the WJ-MSC-EXO treated group and the combination of exosome and Met were reported to be 720.36 ± 62.21 pg/ml (P = 0.0005) and 542.36 ± 23.985 pg/ml (P < 0.0001), respectively (Fig. 6A).

The mean concentration of IL-6 in the 12-h supernatant in the control group treated with LG DMEM medium was 681.23 ± 36.28 pg/ml, which increased to 2569.32 ± 58.65 in the group treated with HG DMEM medium (P < 0.0001). The production of IL-6 in the group treated with HG DMEM and metformin was 1430.47 ± 41.87 pg/ml, which was significantly reduced compared to the group treated with HG DMEM (P < 0.0001). Also, the concentration of this cytokine in the group treated with DMEM high glucose and MSC-EXO and the combination of MSC-EXO and metformin were reported 1933.96 ± 21.395 pg/ml (P < 0.0001) and 540.69 ± 35.359 pg/ml (P < 0.0001), respectively (Fig. 6B).

It was also seen that the mean concentration of TNF-α in the LG DMEM medium cultured HepG2 cell supernatant was 420.36 ± 20.250 pg/ml, which increased to 981.362 ± 78.69 pg/ml in the presence of HG culture medium (P = 0.0003). TNF-α production in Met-treated cells was 548.356 ± 51.065 pg/ml (P = 0.0013). Also, the production of this cytokine in the exosome and the combination-treated group were reported at 583.741 ± 81.78 pg/ml (P = 0.0036) and 374.562 ± 31.9 pg/ml (P = 0.0002), respectively 12-h after treatment (Fig. 6C).

According to the results, the mean concentration of IL-10 in the 12-h supernatant in the control group treated with LG DMEM medium was 1545.367 ± 62.098 pg/ml, which decreased to 856.632 ± 62.781 in the HG DMEM medium treated group (P = 0.0002). The production of IL-10 in the HG DMEM and the metformin-treated group was 941.342 ± 41.786 pg/ml (P = 0.1204). Also, the concentration of this cytokine in the group treated with DMEM high glucose and MSC-EXO and the combination of MSC-EXO and metformin were reported to be 1192.651 ± 46.065 pg/ml (P = 0.0017) and 1675.357 ± 45.98 pg/ml (P < 0.0001), respectively (Fig. 6D).

### 3.5 Cytokines mRNA expression

**Fig. 7:**
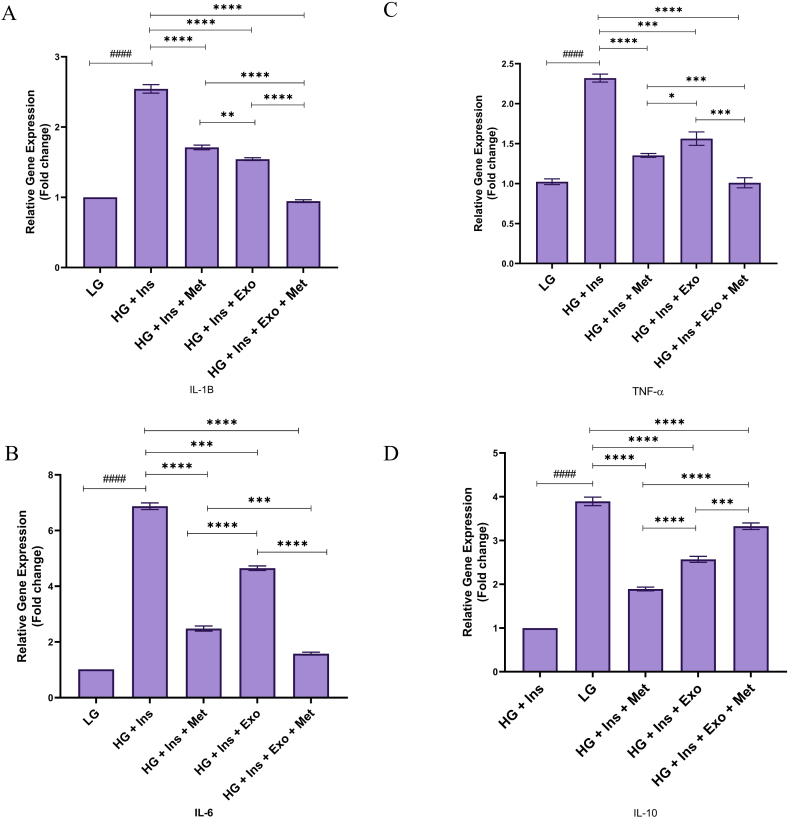
**mRNA expression of inflammatory/anti-inflammatory cytokines.** The relative expression of these cytokines in the different experimental groups was measured using RT-PCR 12 h after treatment. Statistical analysis was performed by one-way ANOVA followed by Tukey's multiple comparison test. Results were presented as means ± standard deviation (SD). * indicates a P value of *<* 0.05, ** indicates a P value of *<* 0.01, *** indicates a P value of <0.001, and **** indicates a P value of <0.0001.

The relative expression of IL-1β in the HG DMEM cultured HepG2 cells is shown in Fig. 7A. The IL-1β expression in HepG2 cells FC increased to 2.543 ± 0.063 (P < 0.0001) 12 h after exposure to HG medium. The IL-1β mRNA expression FC in the treatment group with metformin, exosome, and combination was recorded at 1.71 ± 0.033 (P < 0.0001), 1.542 ± 0.019 (P < 0.0001) and 0.945 ± 0.024 (P < 0.0001), respectively.

12 h after incubation, IL-6 mRNA expression in HG-treated groups increased significantly compared to the LG DMEM cultured HepG2 groups (Fig. 7B). The highest level of IL-6 mRNA expression was in HG-treated HepG2 cells (6.872 ± 0.12, P < 0.0001). The IL-6 mRNA expression in the combination-treated group was the lowest among HG-treated groups (1.58 ± 0.052, P < 0.0001).

The relative expression of the TNF-α as an inflammatory cytokine in the control group cultured by LG DMEM medium is shown in Fig. 7C. The level of TNF-α FC in HepG2 cells increased to 2.326 ± 0.054 after treatment with HG DMEM medium and insulin. The levels of TNF-α-related FC in the metformin treatment group, MSC-EXO, and combination therapy were reported to be 1.354 ± 0.0223, 1.564 ± 0.086, and 1.01 ± 0.063, respectively.

The IL-10 mRNA expression in the combination therapy group, unlike other cytokines expression patterns, is more than HG treated group. The relative expression of IL-10 as an anti-inflammatory cytokine in the control group cultured by LG DMEM medium is shown in Fig. 7D. The level of IL-10-related FCin HepG2 cells decreased to 0.099 ± 0.056 after treatment with HG DMEM and insulin. IL-10 FC levels in the metformin-treated group, MSC-derived exosome treatment, and combination therapy groups were 1.892 ± 0.043, 2.569 ± 0.069, and 3.327 ± 0.074, respectively (P < 0.0001). It was reported that the highest therapeutic effects and increased cytokine IL-10 were observed in the combination therapy group.

### 3.6 Evaluation of cell viability

**Fig. 8:**
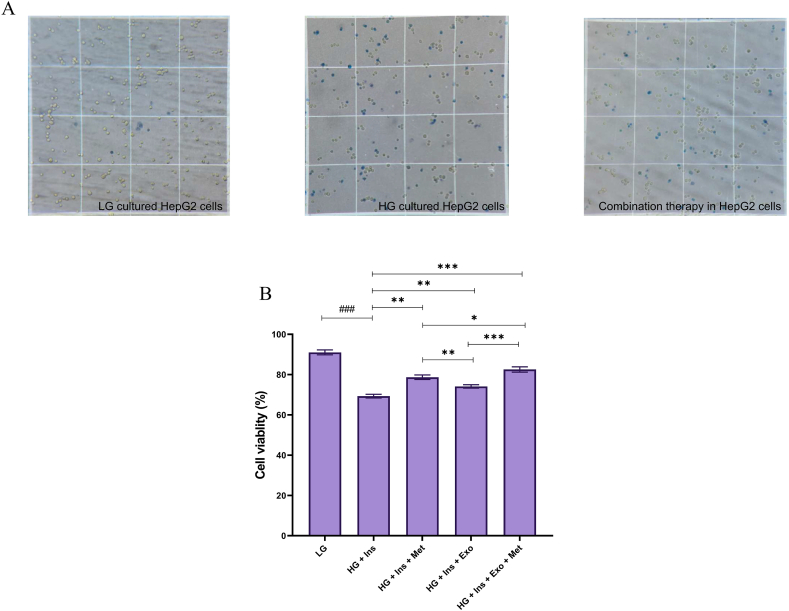
MTT assay for HepG2 cells viability.

The results of the MTT test showed that the viability of HepG2 cells in the combination therapy by metformin and MSC-EXO was higher than the other experimental groups (82.54 ± 1.31, P < 0.001). Also, trypan blue staining showed that this group's cell survival rate was higher than in other experimental groups (Fig. 8A and B).

### 3.7 Glucose consumption in experimental groups

**Fig. 9:**
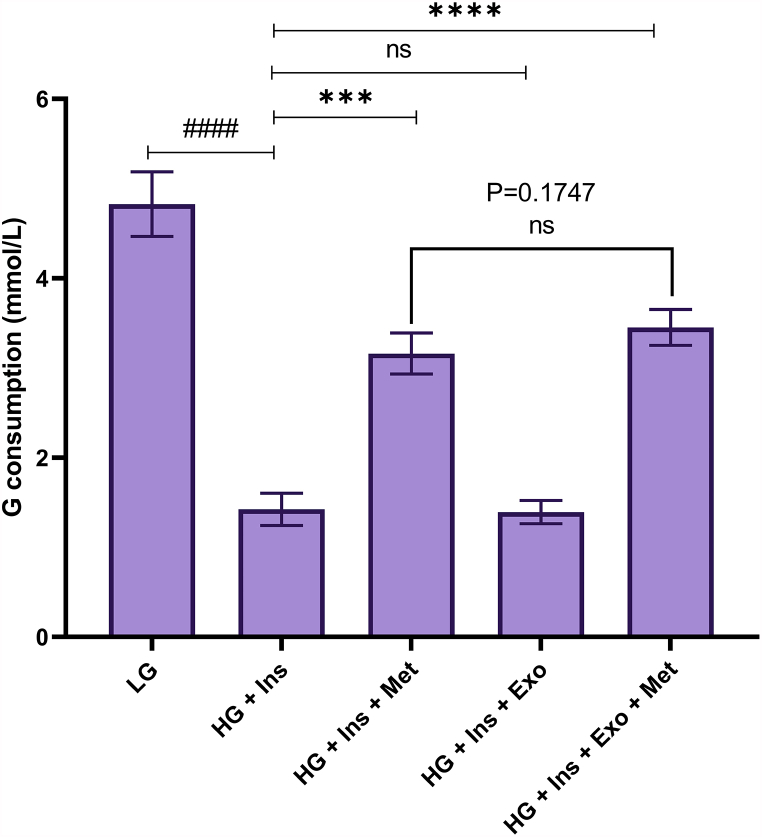
Evaluation of glucose consumption in experimental groups.

Due to the effect of metformin on gluconeogenesis and glucose uptake in liver cells, the supernatant was collected after 12 h of treatment and tested for glucose in the supernatant. The results showed that glucose consumption in the metformin-treated (3.161 ± 0.23 mmol/L, P < 0.001) and combination therapy groups (3.451 ± 0.2 mmol/L, P < 0.001) was significantly increased compared to the HG DMEM and insulin-treated groups. However, MSC-EXO alone has no significant effect on glucose consumption compared to HG-treated HepG2 cells (1.392 ± 0.13 mmol/L, P = 0.8516). (Fig. 9).

### 3.8 Evaluation of apoptosis

**Fig. 10:**
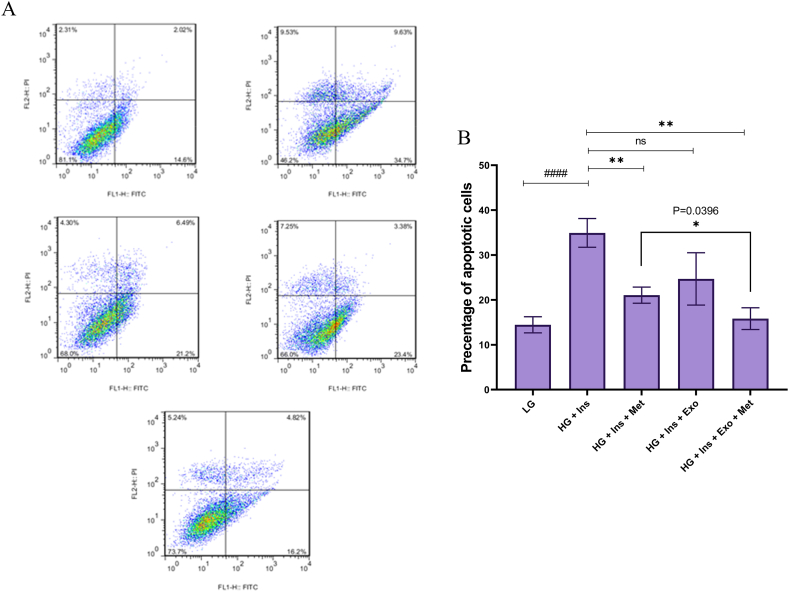
**Evaluation of the effect of treatments on the rate of apoptosis in E.coli infected HepG2 cell by Annexin V-PI staining.** (A) Dot plot diagrams show the percent of necrotic cells (Q1), late apoptotic cells (Q2), early apoptotic cells (Q3), and viable cells (Q4). (B) The bar chart shows the mean percentage of apoptosis. Results were presented as means ± standard deviation (SD). * indicates a P value of <0.05, ** indicates a P value of <0.01, *** indicates a P value of <0.001, and **** indicates a P value of <0.0001.

Flow cytometry evaluated apoptosis in HepG2 cells 12 h after treatment. The percentages of viable and apoptotic cells were reported in Fig. 10A and B. The mean percentage of apoptosis in HepG2 cells was 14.43 ± 1.8%, which reached 34.93 ± 3.2% in HG DMEM-treated group (P = 0.0006). The mean percentage of apoptosis in the Met treated group, WJ-MSC-EXO treated group, and combination therapy were 21.06 ± 1.83% (P = 0.0029), 24.68 ± 5.83% (P = 0.0558), and 15.82 ± 2.42% (P = 0.0012), respectively. As shown in Fig. 10B, the apoptosis rate in the combination therapy group was associated with a 46.321% decrease compared to the HG DMEM and insulin treatment group, which is statistically significant (P = 0.0396).

## 4 Discussion

Due to the importance of inflammation in the pathogenesis of different diseases, including T2D, atherosclerosis, Crohn's disease, rheumatoid arthritis (RA), and cancer, over the past decade, many studies have focused on this issue [26]. Activation of the immune system is closely linked to the onset and progression of T2D, and immune system cells are involved in the pathogenesis of the disease [27].

The liver plays an essential role in inflammation through the production of inflammatory cytokines and acute phase proteins (APP) [24,28]. In addition to liver resident immune cells, it has also been reported that when inflammatory mediators or fatty acids stimulate hepatocytes, they can secrete pro-inflammatory cytokines [29,30]. It was also seen hepatocytes can recognize LPS by TLR4 and activate to protect the liver from bacterial infection [31].

Blood glucose level has been shown to be a key activator of altered metabolic and immune signals in diabetes, leading to the induction of inflammation in liver cells, including hepatocytes [32]. However, the pathway of high glucose-induced inflammatory responses in liver cells is poorly understood [33]. The results of Parsalar et al.'s study indicate that the use of high glucose-containing medium cell culture, causes inflammation and production of pro-inflammatory cytokines in HepG2 cells, which may be due to the production of ROS and activation of MAPKs signaling pathways as well as NF-kB transcription factor activation [34,35].

Several important pathways are known to cause liver damage in T2D [36]. Insulin resistance is the main cause of compensatory hyperinsulinemia, and hyperglycemia is the leading cause of liver damage in diabetic patients [37,38]. The liver, as an insulin-sensitive tissue, is one of the primary organs that are prone to the effects of oxidative stress caused by high blood glucose levels, which may lead to liver tissue damage [39]. Oxidative stress and inflammatory responses induced by immune system cells and liver parenchymal cells (such as hepatocytes) disrupt the metabolism of proteins, carbohydrates, and lipids [40]. As a result, positive feedback leads to increased oxidative stress and the formation of inflammatory cascades. In pathological conditions such as T2D, oxidative stress, and inflammatory responses act as damaging factors and aggravate disease progression [41,42].

T2D treatment is a challenging issue for the global community. Today, researchers are studying safe and effective drugs to overcome the destructive effects of insulin-related metabolic disorders, including hyperglycemia, hyperinsulinemia, hyperlipidemia, oxidative stress, inflammation, atherosclerosis, and other complications. Meanwhile, until now, there is still no specific treatment for patients with NAFLD and NASH besides diet and lifestyle modifications to lose weight and prevent further injuries [43]. However, combination therapy is used to improve insulin sensitivity in the liver (metformin and pioglitazone) [44] and surrounding thiazolidinediones along with other drugs such as betaine, atorvastatin, losartan, and orlistat [[45], [46], [47]]. Based on previous studies, metformin can improve insulin sensitivity by increasing insulin receptor activity. Other effects of metformin may result from its effect on the hyperglycaemic states related membrane fluidity and improve its functions. Therefore metformin improves peripheral and hepatic insulin sensitivity, with direct and indirect impacts on the liver [48]. However, the clinical outcome of these treatments depends very much on the patient receiving the medication and the disease condition. Patients taking these drugs should be closely monitored during detoxification due to possible contraindications to T2D and other medicines [49]. Given the role of inflammatory responses in the development of diabetes-related liver damage, we use an anti-inflammatory agent n combination with metformin, which is the first line of treatment for diabetes, to induce anti-inflammatory responses in HepG2 cells.

MSCs-derived exosomes mimic the characteristics of their origin cells and have anti-inflammatory and immunomodulatory properties [50]. Exosomes have lower membrane protein, are less complex, and are smaller in size compared to their parent cells. Also, exosome isolation and storage and have lower immunogenicity (high biocompatibility) compared to cell therapy [14]. Mesenchymal stem cells derived exosomes perform their therapeutic effects by suppressing oxidative stress, stimulating cell proliferation, regulating the immune system responses, preventing apoptosis, and increasing angiogenesis [51]. These exosomes transport active enzymes to help tissue homeostasis and retrieve normal cell activity [52]. MSCs-derived exosome proteomic analyses show more than 200 immunomodulatory molecules transferred by them to target cells [53]. MSCs-derived exosomes contain various growth factors, anti-inflammatory cytokines, micro-RNAs that modulate inflammation responses, enzymes involved in restoring cell functions, proteins that inhibit apoptosis, and proteins involved in cell metabolism [54]. Also, exosomes can carry hole mitochondria or different components of mitochondria [55]. Transferring mitochondria to target cells can change their metabolic properties, proliferation, differentiation, and inflammatory responses [55]. Also, it has been shown that MSCs-derived exosomes increase proliferation in the target cell and prevents apoptosis by activating and regulating the cells' main signaling pathways, including PTEN/PI3K/AKT/mTOR and Ras/Raf/MEK/ERK [56].

Compared with either treatment alone, the combined use of metformin and mesenchymal stem cell-derived exosomes reduces the production of cytokines IL1-β, IL-6, and TNF-α pro-inflammatory. Also, it has been shown the relative gene expression of pro-inflammatory cytokines such as IL1-β, IL-6, and TNF-α, and apoptosis reduced in insulin resistance-induced HepG2 models after treatment with a combination of metformin and MSC-EXO compared to other groups. According to the results of this study, in contrast to inflammatory cytokines, the concentration and relative gene expression of IL-10 as an anti-inflammatory agent is increased in combinational use of metformin and MSC-EXO, compared with the use of each of these therapies alone.

Therefore, it was shown that using MSCs-derived exosomes as an anti-inflammatory agent combined with metformin can increase the therapeutic efficacy of metformin without changing metformin doses. However, this research is conducted in the In-vitro condition and confirms the effectiveness of combination therapy, and more studies are needed to prove their effectiveness in the clinic.

## Author contribution statement

Kosar Malekpour, Ali Hazrati: Wrote the paper; Analyzed and interpreted the data; Performed the experiments.

Sara Soudi, Leila Roshangar: Conceived and designed the experiments; Analyzed and interpreted the data.

Ali Akbar Pourfathollah, Majid Ahmadi: Contributed in providing reagents, materials, analysis tools and data; Conceived and designed the experiments.

## Data availability statement

Data will be made available on request.

## Additional information

No additional information is available for this paper.

## Availability of data and materials

The data supporting the conclusions of this article are all online.

## Declaration of interest's statement

The authors declare no conflict of interest.
